# Augmenting genetic algorithms with machine learning for inverse molecular design

**DOI:** 10.1039/d4sc02934h

**Published:** 2024-09-11

**Authors:** Hannes Kneiding, David Balcells

**Affiliations:** a Hylleraas Centre for Quantum Molecular Sciences, Department of Chemistry, University of Oslo P.O. Box 1033, Blindern 0315 Oslo Norway david.balcells@kjemi.uio.no

## Abstract

Evolutionary and machine learning methods have been successfully applied to the generation of molecules and materials exhibiting desired properties. The combination of these two paradigms in inverse design tasks can yield powerful methods that explore massive chemical spaces more efficiently, improving the quality of the generated compounds. However, such synergistic approaches are still an incipient area of research and appear underexplored in the literature. This perspective covers different ways of incorporating machine learning approaches into evolutionary learning frameworks, with the overall goal of increasing the optimization efficiency of genetic algorithms. In particular, machine learning surrogate models for faster fitness function evaluation, discriminator models to control population diversity on-the-fly, machine learning based crossover operations, and evolution in latent space are discussed. The further potential of these synergistic approaches in generative tasks is also assessed, outlining promising directions for future developments.

## Abstract 2

Evolutionary and machine learning methods have been successfully combined and applied to the generation of molecules and materials exhibiting desired properties.

## Graphical abstract

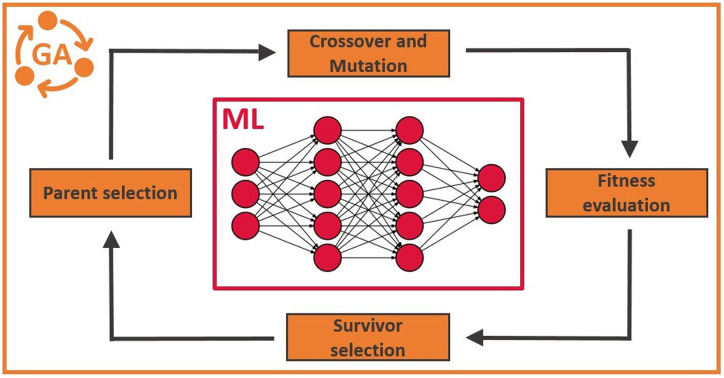

## Introduction

One of the main goals in materials science is the discovery of new chemical compounds that exhibit certain properties that make them optimal for specific applications. There is a constant demand for such new and improved materials in many different research areas and examples include the fields of drug design,^1–4^ catalysis,^5–10^ and battery research.^11–13^ The virtually infinite size of the chemical space however, makes an exhaustive search impossible and dictates the use of efficient optimization methods that suggest candidate compounds by leveraging existing knowledge about the domain of interest. These generative models tackle the inverse design problem, where the objective is to find solutions that optimally satisfy a set of requirements imposed by a given specification.^14^ Evolutionary approaches in particular, are inspired by Darwinian evolution and operate on a population of solutions that is evolved in order to incrementally produce solutions that better fit these requirements. In chemistry and materials science, evolutionary approaches have been adopted already early in the 1990s,^15^ for example in the *de novo* design of polymers,^16^ proteins^17^ and refrigerants.^18^ With the explosion of (deep) machine learning in the 2010s, these endeavors have somewhat been neglected in favor of other generative methods based on artificial neural networks (ANNs) such as recurrent neural networks,^19–21^ variational autoencoders,^22–24^ normalizing flows,^25–27^ and diffusion models.^28–30^ Nonetheless, these models often times fall short in real world applications because they do not include relevant constraints like limited training data and computational resources or the synthetic accessibility of the generated molecules.^31^ Evolutionary approaches, on the other hand, require only little initial data, exhibit higher computational efficiency^32^ and their optimization aim can be easily modulated to incorporate additional constraints. They furthermore have the ability to explore truly new regions of chemical space whereas ANN-based approaches tend to be limited to molecules that are similar to the training set. There recently has been an uptake in interest for evolutionary optimization in chemistry, with successful applications to diverse problems including the design of mechanosensitive conductors,^33^ organic emitters,^34^ polymers,^35–37^ drug-like molecules,^38–41^ and catalysts.^42–46^

Given the success of both evolutionary and machine learning in materials science, it is natural to investigate the combination of both approaches. While being an incipient area of research still in its infancy, efforts have been made to explore the synergies and some very promising advances have already been achieved.

In this perspective we will first give a brief introduction to evolutionary learning (EL) and in particular genetic algorithms (GAs). Next, we will review a series of studies on materials optimization using hybrid approaches that utilize techniques from evolutionary and machine learning. Finally, we conclude with a short summary and discuss opportunities for further developments and applications.

GAs, first popularized by Holland in the 1970s,^47^ are one of many different types of EL algorithms and are commonly used in materials science for the *de novo* design of materials and molecules.^48^ Like all other types of EL approaches, they are generic and heuristic optimization algorithms that make no prior assumptions about the solution domain. GAs are inspired by Darwinian evolution and draw concepts from evolutionary biology such as mutation, recombination, and selection. The underlying key idea of GAs is that evolution is dictated by two competing forces: variation, which pushes the population towards novelty, and selection, which pushes the population towards quality. Combining both forces in an iterative optimization scheme leads to an efficient search strategy that balances exploration and exploitation in solution space. The efficiency of GAs is due to the heuristic nature of selection and recombination operations, that leverage the best partial solutions to construct better solutions. This makes them ideal for exploring chemical spaces that are usually large and diverse. On the flip side however, this also means that GAs are non-deterministic, meaning that they are not guaranteed to converge to the global optimum.

In the following, the basic building blocks of GAs are briefly described. The literature provides comprehensive overviews and discussions on the essential building blocks of GAs^49^ and their applications to chemistry and materials science.^48^

GAs operate on a set of solutions called the population, that is iteratively optimized to yield higher quality solutions over the course of multiple generations. Following the language of evolutionary biology, the solutions are also called individuals, which in chemistry and related fields usually represent molecules or materials.^48^ In each generation, new offspring solutions are created by applying genetic operations that combine information of the currently best performing solutions (exploitation) and introduce random mutations (exploration). The newly generated offspring solutions then compete against the solutions of the previous population, and only the best performing solutions are carried over to the next generation. This process is repeated until some sort of convergence criterion is met (often times simply a maximal number of iterations).^49,50^ There are four main building blocks to any GA that can be adapted in order to modify the evolution behavior in terms of the search space, optimization target, selection pressure, and diversity control:

• Chromosome: defines the representation of the solutions.

• Fitness: measures the quality of the solutions.

• Genetic operations: create new solutions from existing ones.

• Selection: selects individuals of the population based on their fitness.

This modular nature makes GAs ideal for applications in chemistry and materials science where optimization tasks are usually problem specific and diverse.^48^ All solutions in a GA share a common, underlying structure that completely defines their traits. In technical terms, this is represented as an array where each cell corresponds to a different property of the solution. These cells are referred to as the genes, which in the array, form the chromosome, expressing properties of a problem-dependent nature. The chromosome usually has a fixed length and its cell values can be of different data types (*e.g.* boolean, integer or float). During evolution, new offspring solutions will be created by applying genetic operations to the chromosome. Therefore, all chromosome values are usually constrained to be of the same data type so that meaningful recombination operations between them can be defined.^49,50^ In applications to chemistry and material science the chromosome is the molecular representation.^48^ Most commonly used are line notations such as SMILES^51^ that represent molecules using a single cell of data type string.

The quality of a solution is measured in terms of a so-called fitness that reflects how well it satisfies a specified set of requirements. Thereby, it essentially defines the optimization objective and is determined by the specific problem to be solved. The fitness is a real valued function of the chromosome that can be thought of as a hyper-surface on which the GA tries to find (local) optima. In multi-objective optimization settings^52–61^ it is a vector, where each dimension corresponds to a different property of interest. Calculation of the fitness is usually the computational bottleneck of GAs and since it is evaluated multiple times per generation, its choice has significant implications on the overall computational cost and performance.^49,50^

Genetic operations are used to generate new offspring solutions in each generation and push the population towards novelty. They can be subdivided into two groups, crossover and mutation, which are usually performed in sequence. First, the genomes of two parent solutions are recombined in a crossover operation to form an offspring solution that then is further modified by a random mutation. However, there also exist variations to this process in which either crossover or mutation operations alone are used in order to obtain offspring solutions.^49,50^ The crossover propagates characteristics of the parent solutions to the offspring. Together with parent selection, it ensures that genetic information of well performing solutions is carried over to the next generations. There exist different implementations, such as the single point crossover in which the two parent chromosomes are split at a random position and then exchange genes.^49,50^ Mutations, on the other hand, usually introduce completely new genetic information in a random fashion, which ensures diversity in the explored solutions. There are many different implementations for such mutation operations, one example is the single point mutation that randomly changes a single gene in a solution's chromosome. Mutations can also be defined according to a predefined rule, for example the swap mutation switches the values of two genes.^49,50^

Selection pushes the population towards quality by discarding badly performing solutions. Selection is performed twice in each generation, once to determine which solutions are recombined to create new offspring (parent selection), and once to determine which solutions proceed to the next generation (survivor selection). The selection rules are usually stochastic and dependent on the solutions fitnesses so that the fittest are more likely to be selected. This ensures that the population evolves towards better performing solutions while maintaining some level of diversity.^49,50^

In chemistry and materials science, GAs are known to be efficient optimization tools, aiding the exploration of large chemical spaces of diverse nature.^57,62–66^ An important contribution to the field is the graph-based GA (GB-GA)^67^ that utilizes the graph representations of molecules to define crossover and mutation operations offering an alternative to the more common string based SMILES representation.^51^ Recent advances include the PL-MOGA^61^ that facilitates the directed, multi-objective optimization of transition metal complexes, and GAMaterial^68^ which enables the machine learning (ML) accelerated optimization of materials and others.^34,55,69–71^ Furthermore, GAs have been used for the optimization of molecular structures and conformer search. For example in the automated interaction site screening (aISS)^72^ approach, that finds accurate aggregate geometries, such as dimers, at low computational cost.

## Surrogate fitness functions

When evolving molecules and materials, the fitness function is often times expensive and difficult to evaluate. This can be due to the fact that values have to be determined experimentally, which can be challenging in computational approaches, or require doing calculations at an expensive level of theory, such as density functional theory. Therefore, an obvious remedy is to replace the fitness function with a cheaper machine learning model that is fitted to previously existing data. These surrogate models of the fitness^73^ have the ability to drastically reduce the computational cost. Examples of appropriate machine learning methods include but are not limited to linear regression, support vector machines, random forests, and ANNs. The applicability of surrogate models is contingent upon on their predictive accuracy because unreliable fitness values impede the evolutionary optimization progress. Especially for large chemical spaces it can be difficult if not impossible to build a surrogate model with general applicability and sufficient accuracy. This highlights the importance of careful model selection and validation.

Janet and co-workers demonstrated the efficiency of an ANN-based fitness function in the evolutionary optimization of spin-crossover complexes with a characteristic near-zero free energy difference between high (H) and low (L) spin states (*i.e.* the spin splitting energy).^74^ In previous work,^75^ the authors had trained an ANN for predicting spin splitting energies on 2690 relaxed transition metal complexes achieving a root mean squared error of 3 kcal mol^−1^. This prompted the use of these models as a surrogate function in an EL framework for the discovery of spin-crossover complexes. The authors adapted a GA proposed by Shu and co-workers^76^ that models molecules as hierarchical trees where each node represents a molecular fragment and the edges are the chemical bonds connecting them. With a specific set of connection rules, a chemical space of 5664 single-center transition metal complexes could be represented, using 32 diverse, organic ligands. The fitness was modeled with the exponential function1
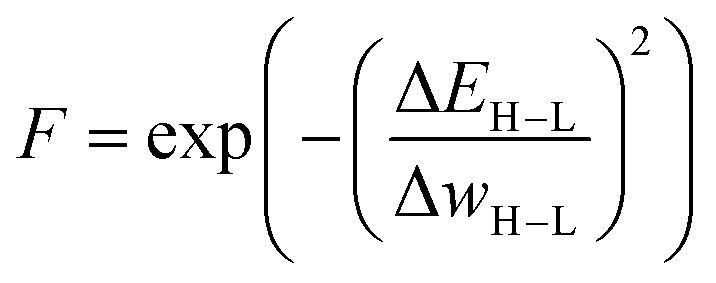
where Δ*E*_H−L_ denotes the spin splitting energy, and Δ*w*_H−L_ denotes a control parameter used to regulate how strongly the fitness decreases for increasing values of Δ*E*_H−L_. Instead of relying on expensive DFT calculations, the spin splitting energies were predicted using the previously trained ANN.^75^ In each generation, parents were chosen by roulette wheel selection with selection probabilities proportional to the absolute fitness values. Crossover operations were defined by an edge breaking operation in the parents and a subsequent exchange of the resulting subtrees. In each generation, five of these crossovers were performed before randomly mutating each tree fragment with a probability of 0.15. The mutation operation replaced the respective fragment with randomly selected fragments that lead to a valid tree according to the connection rules. Survivor selection was done deterministically by choosing the complexes with the highest fitness values from the combined pool of current and new offspring individuals.

**Fig. 1:**
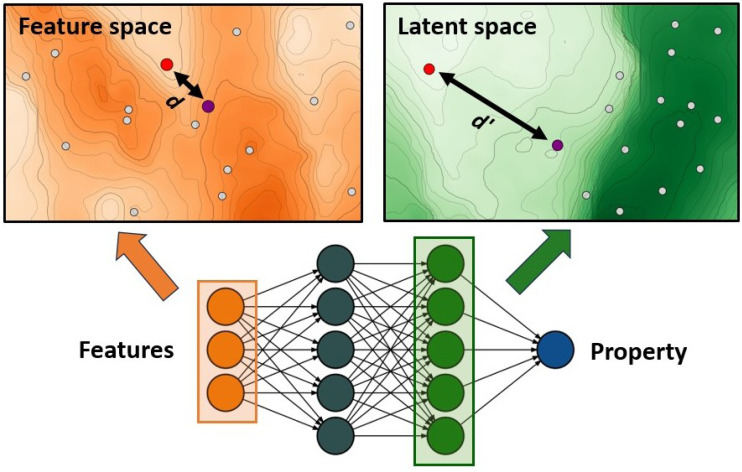
ANN-based surrogate function using the distance in latent space as a measure for uncertainty. Points that are close in feature space might not necessarily be close in latent space.

The authors further proposed two additions to this standard GA framework: a diversity control mechanism to prevent evolutionary stagnation and a distance penalty to account for low prediction confidence of the ANN for data points very different from the training data. Their proposed diversity control mechanism increases the mutation probability to 0.5 if the ratio of unique complexes in the current generation falls below 25%. The increased mutation rate pushes the GA to explore new regions in the chemical space and thereby prevents the GA from getting stuck in a local optimum. The distance penalty is motivated by the observation that ML prediction results tend to become unreliable for data points very different from the training data. In a GA where the fitness is based on surrogate predictions, poor predictive performance can hinder evolution and lead to poor final results. Therefore, using model uncertainty to estimate the surrogate accuracy can be useful to avoid basing evolution on overconfident fitness predictions.^77^ In previous work,^75^ the authors showed that a large distance in feature space is a potent indicator of model accuracy (Fig. 1) and successfully used it with a set of features that emphasizes metal-proximal properties.

This approach was employed here: in order to discourage sampling of candidates with large distances to the training data, the authors introduced a modified fitness function2
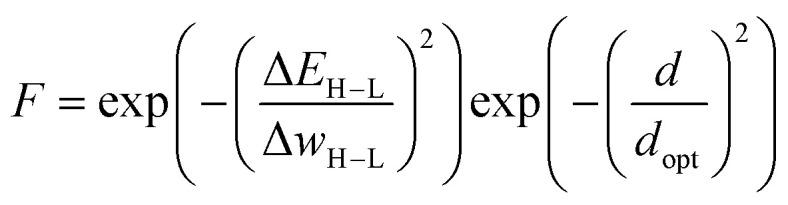
where, the second term is an exponential penalty term with *d* denoting the candidates average distance to the training data points in the MCDL-25 descriptor space,^75^ and *d*_opt_ denoting a control parameter used to scale the distance penalty. In later works,^78^ the authors propose an alternative approach that utilizes the distance in latent space (Fig. 1) instead of feature space, which has the advantage of being less sensitive to feature selection.

They benchmarked four different variants of the GA: (1) the standard GA, (2) GA with diversity control, (3) GA with distance penalty, and (4) GA with both diversity control and distance penalty. In all cases, the GA was initialized with a random selection of 20 complexes and run for a total of 21 generations. The standard GA quickly converged due to a single candidate completely dominating the solution. The GA with diversity control exhibited a slightly higher diversity in the final population while approaching fitness values to those of the standard GA. However, both the standard GA and the diversity-controlled GA converged towards candidates with on average large distances to the training data and therefore low prediction confidence. Introducing the distance penalty term in the fitness function lead to candidates with 50% lower mean distances to the training data at the cost of a 25% reduction of the mean population fitness. With this approach, the authors could achieve both higher diversity in the final candidates as well as fairly small mean distances to the training data. With ∼50 repeats of the GA, roughly half of the design space could be sampled using the standard and diversity-controlled approach. With the combined control strategy, 80% of the lead compounds could be identified, which constitutes an increase of ∼30% compared to the standard GA. The majority of missed lead compounds had large distances to the training data, indicating that the distance penalty term works as intended and discourages exploration in areas of low confidence, which nonetheless contain a small proportion of the leads.

In order to estimate the robustness of the ANN-based fitness, the authors determined the accuracy of the surrogate model for a subset of lead compounds identified by the GA. Relative to DFT, they obtained an average test error of 4.5 kcal mol^−1^, which is moderately higher than the model baseline error of 3.0 kcal mol^−1^. For complexes that were very similar to the training data, the observed mean error was 1.5 kcal mol^−1^. Furthermore, two thirds of the ANN lead compounds could be validated by DFT optimization, though including solvent effects and thermodynamic corrections reduced this ratio to one half. According to the authors, these findings demonstrated sufficient predictive performance of the ANN fitness for its use in evolutionary design applications.

In their conclusion, the authors emphasized the massive gains in terms of computational efficiency compared to a traditional GA with a DFT-based fitness function that would require up to 30 days of computing walltime. However, the computational cost associated with acquiring the training data (here roughly 50% of the investigated space) remains a significant contribution. They furthermore noted that the observed ANN errors in the final populations could be reduced by decreasing *d*_opt_ and discussed options for leveraging low-confidence candidates to retrain the surrogate model on-the-fly, in order to improve the predictive accuracy of the model in subsequent generations.

Forrest and co-worker^79^ made use of similar concepts for the evolutionary optimization of alloy compositions with respect to their glass forming ability. Instead of a single ANN, they used an ensemble of ANNs as a surrogate fitness function in order to facilitate predictions of relevant properties such as the temperature of the crystallization onset. Further, Kwon and co-workers^80^ utilized a surrogate model in an evolutionary approach to optimize the maximum light-absorbing wavelengths in organic molecules. Since their evolutionary algorithm operated directly on bit-string fingerprint vectors^81^ they furthermore used a separate RNN to decode them into chemically valid molecular structures.

## Bayesian surrogate models

**Fig. 2:**
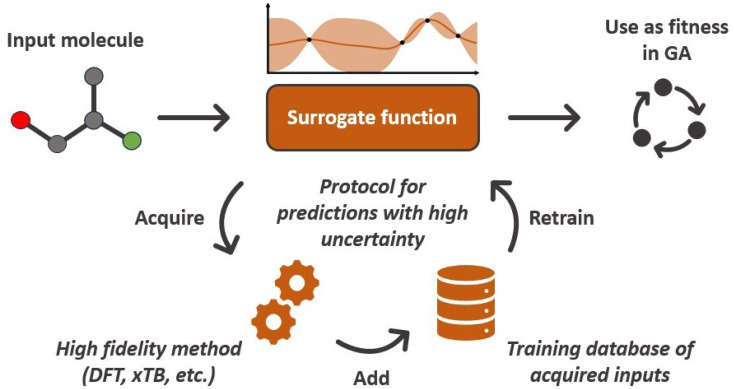
Conceptual workflow of a Bayesian surrogate fitness functions. Data points with high prediction uncertainties are acquired using a high fidelity reference method and added to the training dataset.

A common issue with machine learning surrogate fitness functions is that the initial data that the model is trained on, might not cover the whole chemical space the GA tries to explore. This will lead to low predictive quality which, in turn, hinders the evolutionary progress and causes overall poor results. As in Janet and co-workers work^74,75^ this can be accounted for in the fitness function by discouraging exploration of solutions that are very different from the corresponding training data. An alternative approach to this problem is the so-called active learning framework in which new training data is acquired on-the-fly from a reference function in order to subsequently refit the surrogate model to this extended dataset. In order to minimize the number of times the expensive reference function has to be evaluated, the data points to be acquired should be selected with care. One possible approach for this is to use a Bayesian machine learning model that additionally gives an uncertainty estimate, quantifying the trust the model has in its prediction. If the uncertainty for a given data point is higher than a specified threshold, it should be acquired with the reference function and added to the training data. This ensures that no unnecessary reference function evaluations are performed and efficiently generates a dataset that covers the chemical space of interest. While Bayesian learning methods are the most straightforward way of obtaining uncertainties other approaches for uncertainty estimation exist and often times bear the advantage of lower computational cost.^78^ The general active learning workflow in the context of evolutionary learning is illustrated in Fig. 2. While the active learning framework has been thoroughly explored for applications in chemistry and materials science,^82–86^ its combination with GAs is still a fairly new and unexplored area of research.

In their 2019 study,^87^ Jennings and co-workers showcased such a model by investigating the atom ordering of a 147-atom Mackay icosahedral structure.^88^ They considered all possible compositions Pt_*x*_Au_147−*x*_ for all *x* ∈ [1, 146]. The optimization goal was to locate the hull of minimum excess energy configurations for all compositions as calculated by the Effective Medium Potential (EMT) potential,^89^ which served as the fitness. The authors defined a traditional GA that operates on the configuration of Pt and Au atoms. Cut and splice crossover functions^90^ as well as random permutation and swapping mutations were used to create new offspring configurations. The crossover and mutation operations were set up to be mutually exclusive, meaning that offspring was created using either one or the other method. Parents were selected with a roulette wheel selection scheme based on the fitness values. In order to ensure that all compositions were searched, the authors furthermore employed a niching scheme in which solutions are grouped according to their composition. Their fitness was then determined per niche and the best configurations per composition niche were given equal fitness.

For the surrogate model they employed Gaussian process (GP) regression, the most commonly used method in Bayesian optimization. They employed the squared exponential kernel defined as3
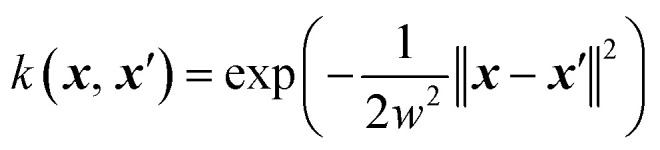
where ***x*** and ***x***′ denote the feature vectors to compare, ‖·‖^2^ denotes the Euclidean distance, and *w* is a hyperparameter defining the kernel width. The inputs to the model were numerical fingerprints that described the chemical ordering within a composition based on the number of nearest neighbors. In particular the feature vector for each configuration was given by4
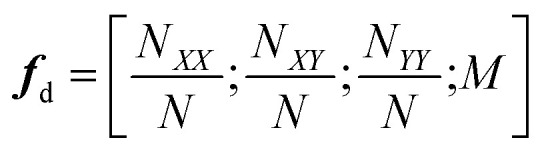
where *N* denotes the number of atoms, *M* denotes the overall mass, and *N*_*XY*_ denotes the number of bonds between atom types *X* and *Y*. The model was trained on relaxed structures even though predictions used the unrelaxed structures. The authors justified this by the fact that their set of descriptors is invariant to small changes in the geometries.

The authors began by setting up a baseline run using a traditional GA using the EMT potential as a fitness function and no surrogate model. With roughly 16 000 fitness evaluations the GA was able to locate the convex hull, which is already a massive improvement compared to the brute-force approach that would require 1.78 × 10^44^ energy evaluations. They continued by setting up an ML accelerated approach based on a nested GA in which the GP-based surrogate fitness function is used. In each iteration the current population in the main GA was passed to the nested GA in which solutions were evolved solely based on the prediction of the GP model trained on the current data. After a number of iterations the evolved population was passed back to the main GA where the true fitness of candidates is calculated with the EMT potential before applying recombination and selection as in the traditional GA. The calculated EMT fitness was furthermore used to retrain the GP model improving its predictive accuracy for the next run of the nested GA. After survivor selection, the population from the main GA was again passed to the nested GA for evolution. The algorithm was terminated when the nested GA did not find any candidates that improved the population. With the ML accelerated GA the authors reported to find the convex hull within 1200 energy evaluations, which constituted about 7.5% of the amount needed in the traditional GA. While in total more structures were checked, most of them were only evaluated using the cheap GP model and only few were calculated with the expensive EMT potential.

The authors furthermore presented this alternative fitness function based on a candidates probability of improving upon the currently best known solution:5
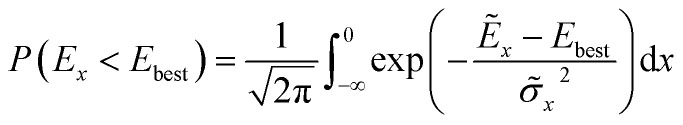
Here, *E*_*x*_ and *E*_best_ denote the EMT energies of the candidate *x* and the currently best known solution respectively, and *Ẽ*_*x*_ and *σ̃*_*x*_^2^ denote the GP-predicted energy and variance, respectively. In this way, the uncertainty of the prediction is included in the fitness function, which encourages the nested GA to also explore unknown regions of the search space. This definition of the fitness is akin to acquisition functions in active learning frameworks, such as the expected improvement score.^91^ Using this approach, the authors reported to find the convex hull with only 280 required energy calculations indicating its superior ability to efficiently sample the solution space.

Finally, the authors replaced the EMT potential with a more accurate DFT calculation and repeated the experiments in order to prove that the obtained results were not an artifact of the EMT potential. The results showed that a performance similar to that observed with the EMT potential could be achieved, requiring ∼700 DFT evaluations.

Overall, this work demonstrated a significant speed-up with their ML accelerated GA and motivated further improvements by proposing a way of including geometry optimization with additional genetic operators acting on the atomic coordinates.

## Ensuring population diversity

Sufficient exploration of the chemical search space is a key challenge when employing GAs for the *de novo* design of molecules and materials. Often times the optimization can get stuck in local optima due to low diversity in the population of solutions, which prevents the GA from exploring all relevant regions in the search space. This leads to slow convergence and overall poor results. Therefore, an efficient, on-the-fly management of the population diversity is essential in order to ensure comprehensive sampling of the chemical space.

To tackle this problem with an ML approach, Nigam and co-workers proposed an augmented GA architecture that includes an ANN with the explicit task of increasing the populations diversity.^92^ They modeled the fitness as a linear combination of the molecular property to optimize (*J*) and a discriminator score (*D*) that measures the novelty of the molecule *m*:

6 *F*(*m*) = *J*(*m*) + *β* × *D*(*m*)

where *β* denotes a hyperparameter that is used to control the weight of the discriminator score and *J* was chosen to be the penalized logarithm of the water–octanol partition coefficient defined as

7 *J*(*m*) = log *P*(*m*) − SA(*m*) − RP(*m*)

where log *P* denotes the logarithm of the water–octanol partition coefficient, which is the actual target, SA denotes a synthetic accessibility penalty,^93^ and RP denotes a penalty for rings with more than 6 atoms. The GA operates directly on the so-called SELFIES^94,95^ strings that represent the different molecules. Compared to the more traditional SMILES strings,^51^ SELFIES are defined in terms of a formal grammar comprising a set of derivation rules. With these, SELFIES can be translated into SMILES character-by-character akin to a state machine, where the next output character depends on the current state of the machine and the input. The set of derivation rules is crafted so that all SELFIES correspond to a valid molecule, making them an extremely robust molecular representation. The authors restricted the search space to solutions that produce SMILES strings with up to 81 characters. The robustness of this representation allowed for specifying random insertion and replacement mutations directly on the SELFIES character level. In contrast to the standard GA setup, they did not use any crossover operations, meaning that offspring solutions were created by only applying these mutations to the parents. Survivor selection at the end of each generation was performed stochastically, where selection probabilities were calculated using a logistic function based on the fitness rankings of solutions.

**Fig. 3:**
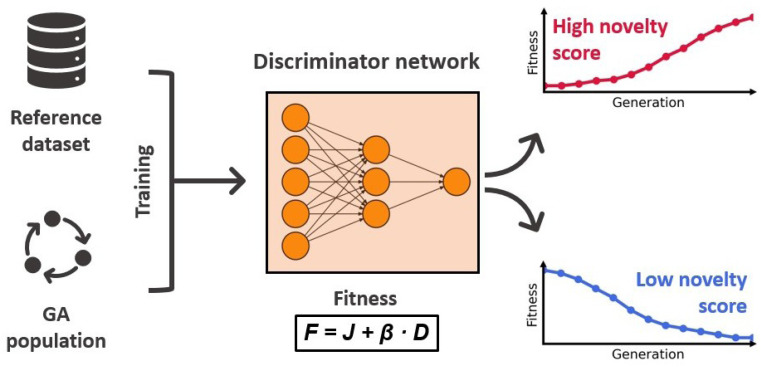
Workflow of the discriminator ANN. The overall fitness *F* is calculated as a weighted sum of the optimization target *J* and the discriminator ANN novelty score *D*. The fitness of long-surviving candidates with low novelty will gradually decrease making their survival less likely.

The discriminator *D*(·) is a dense ANN with ReLU activations and a sigmoid output layer that distinguishes molecules generated by the GA from molecules of a reference dataset. In each generation it is trained for 10 epochs on the molecules of the current population and an equal amount of molecules randomly drawn from the reference dataset, using chemical and geometrical properties as features. In the next generation, this model is then used to assign novelty scores to the newly generated molecules. Molecules that are similar to the molecules of the previous generation will receive low scores, whereas molecules that are more similar to structures of the reference dataset will receive high scores. Because the discriminator score enters the fitness function, the novelty of proposed molecules directly influences their chance of survival. This effect is illustrated in Fig. 3 displaying the workflow of the discriminator ANN. A nice property of this approach is that the discriminator ANN will become very good at identifying long-surviving molecules, assigning low novelty scores, and therefore making it less likely that they will proceed to the next generation. This discourages the survival of already explored solutions and forces the GA to explore regions of the chemical space that are similar to the reference dataset. The authors confirmed this in an investigative study showing that the higher the value of *β*, the more similar to the reference dataset are the proposed molecules.

The authors further refined their approach with an adaptive discriminator scheme that introduces a time-dependence for the *β* parameter. In this setting, *β* is set to zero and only if the optimization stagnates its value is increased to 1000, in order to encourage exploration. Once stagnation is overcome, *β* will be set to zero again.

In their experiments, the authors used a subselection of 250k commercially available molecules from the ZINC dataset^96^ as the reference dataset and benchmarked their architecture with (*β* = 10) and without (*β* = 0) the discriminator module. The results showed an increase in the maximum, achieved, penalized log *P* values of roughly 5% by using the discriminator. The best log *P* values were achieved with the time-dependent discriminator, giving a 55% performance increase compared to the regular discriminator. The authors claimed to outperform the highest literature values by a factor of more than 2. With a principal component analysis and clustering of all generated molecules, the authors furthermore showed that, in the time-dependent approach, the population never stagnated in one chemical family, sequentially moving towards different regions in the chemical space. The study also focused on the simultaneous optimization of log *P* and drug-likeness by incorporating the QED score^97^ in the fitness function. Their benchmark results on the ZINC^96^ and GuacaMol^98^ datasets suggested that their GA is able to efficiently sample the Pareto front spanned by the two properties.

The authors concluded by highlighting the domain independence of their approach, making it interesting also for applications outside the field of chemistry and discussed possible improvements using another ANN for fitness evaluation.

## Balancing exploration and exploitation

While broad exploration of the chemical search space is important for sampling from a variety of different molecular families, effective exploitation for finding the best solutions within these local regions is equally important in order to obtain optimal results. However, increasing selection pressure in order to promote solutions of higher quality often times compromises a GA's explorative ability because suboptimal steps that might be necessary to escape local optima are strongly discouraged. Finding a good balance between exploration and exploitation is crucial in order to maximize the quality of the final population and increase the GAs efficiency.

**Fig. 4:**
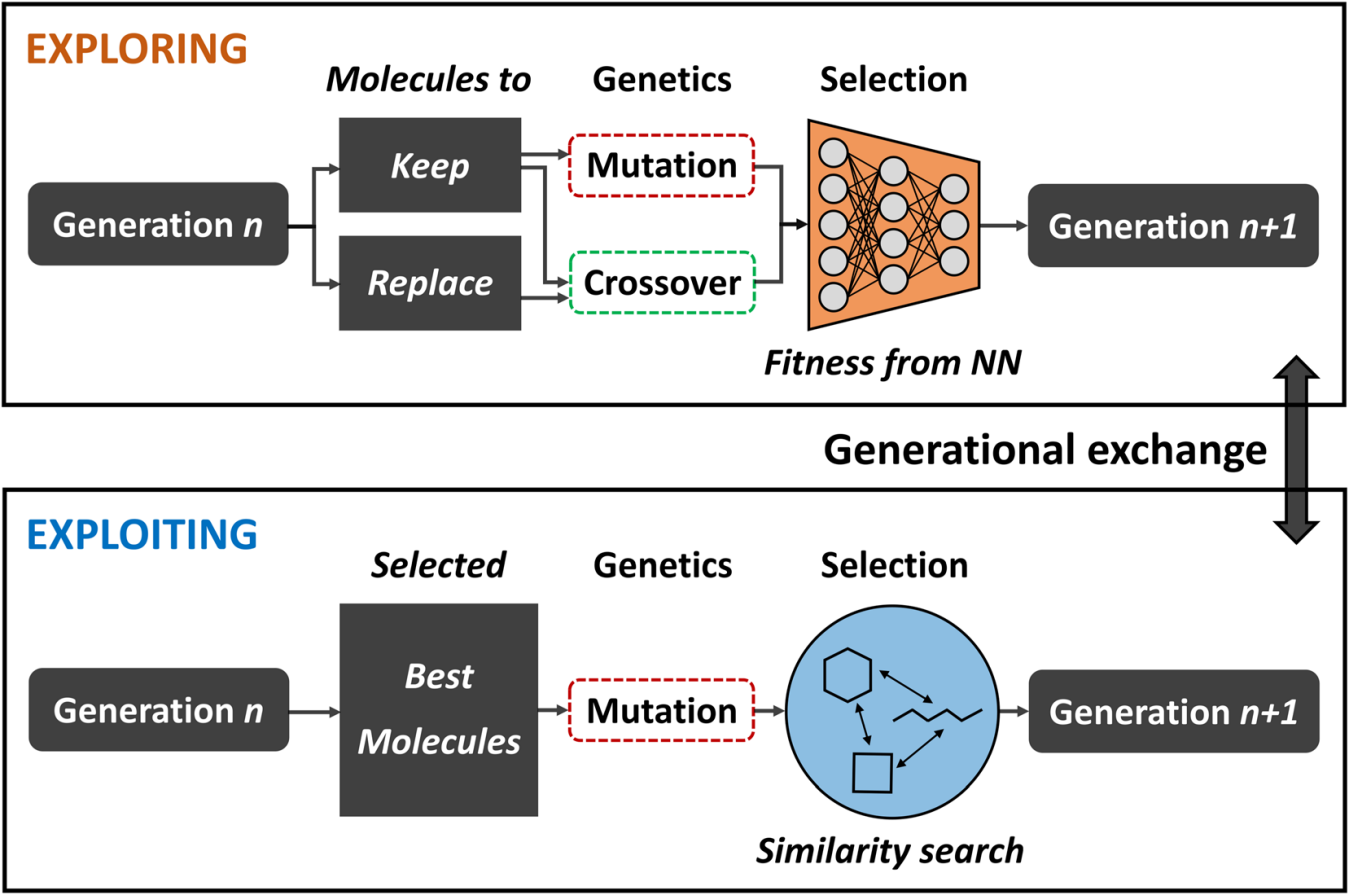
Schematic depiction of the JANUS architecture. Two separate populations are propagated in parallel using different genetic operations in order to promote exploration in one, and exploitation in the other. An exchange of individuals at the end of each generation allows the two populations to mix.

To that end, Nigam and co-workers improved on their previous ANN-augmented GA^92^ by proposing JANUS,^99^ a parallel GA guided by ANNs. JANUS maintains two distinct and independent, fixed size populations as they are separately evolved. With this two-pronged approach, one population is responsible for exploration while the other takes care of exploitation. At the beginning of each generation, the populations can furthermore exchange individuals in order to combine the benefits of both approaches. A schematic description of the JANUS architecture is shown in Fig. 4.

Analogous to their previous work, JANUS operates directly on SELFIES strings that represent molecules and, as in any other GA, the quality of an individual is measured using a fitness function. Selection of survivors is performed deterministically, meaning that only the best solutions proceed to the next generation. The genetic operators employed differ for the two different populations in order to promote either exploration or exploitation. The exploitative population uses the same insertion and replacement mutations excluding crossovers, as in their previous work,^92^ and applies them to the *n* currently best solutions. In addition to these mutations, the explorative population uses an interpolation crossover that generates paths between two parents through matching and replacing characters until both are equal. For all intermediates along these paths, the joint similarity^100^ to both parents in terms of the Tanimoto score is calculated and the molecule with the highest score is selected as the final offspring resulting from crossover. Parents are selected according to a simulated annealing strategy that allows badly performing individuals to be selected with low probabilities which allows the population to escape local optima.

Additional selection pressure is applied to filter the offspring individuals before survivor selection. In the exploitative population only molecules that are similar to the parents are propagated further. In the explorative population an ANN is used to predict fitness values and, based on its predictions, the highest scoring individuals are added to the population. Alternatively, a classifier ANN can be used which directly sorts the offspring individuals into either “good” or “bad”, only allowing “good” molecules to enter the population. Either approach effectively constitutes a pre-selection of the most promising solutions at a low computational cost. The ANN is trained in each generation with all molecules for which the fitness is known. This implies that the ANNs predictive accuracy becomes better over the course of multiple generations as more data is added to the training set. For the classifier ANN, a %-threshold is used to identify which molecules belong to the “good” and “bad” classes. In their study, the authors experimented with the top 50% and 20%.

The authors tested their architecture on common molecular design benchmarks. As in their previous work,^92^ they first investigated the optimization of the penalized log *P* value as defined in eqn (7), modeling molecules using the robust SELFIES representation with a maximum character limit of 81. In total, four different variations of JANUS were tested: plain without additional selection pressure added in the explorative population, modified with the fitness ANN predictor, and modified with the ANN classifier with thresholds of 50% and 20%. All variants outperformed other approaches from the literature in terms of the single best molecule. On average only one model (genetic expert-guided learning^101^) performed better than JANUS. The authors' previous GA discriminator approach^92^ could achieve results of similar quality only after 10 times the number of generations. Using the fitness ANN predictor increased the median population fitness compared to the plain model without additional selection pressure. Similar trends could be observed for the ANN classifiers, all converging into the same local optimum within 100 generations. The convergence rate however, showed a significant dependence on the thresholds. With the 20% threshold, the local optimum was reached already after less than 20 generations, whereas with the 50% threshold, almost 100 generations were required. At the same time, the 20% threshold limited the exploration of the chemical space as indicated by the smaller fitness ranges spanned in the generations. With the 50% threshold, these ranges were much larger even surpassing those obtained with the ANN predictor. Finally, the authors compared the number of evaluations needed in order to reach certain fitness values (*J*(*m*) = {10, 15, 20}) for all four variants. The model using the ANN classifier with a threshold of 20% needed the smallest number of evaluations, and the model using the ANN predictor needed the second smallest. The largest number of evaluations was required by the plain model, highlighting the benefit of the additional selection pressure introduced by the ANNs.

JANUS was furthermore tested on two more molecular benchmarks: the imitated protein inhibition task,^102^ in which the objective is to generate 5000 molecules that inhibit two different proteins (either one or both) while exhibiting high drug-likeness as measured by the QED score^97^ and low synthetic accessibility penalty as measured by the SA score,^93^ and a docking task^103^ considering four different protein targets with the goal of finding molecules that minimize the respective docking scores. In both benchmarks the authors found JANUS to outperform other approaches from the literature and achieve state-of-the-art results in terms of the fitness objective. The diversity of generated molecules however was reduced compared to results from the literature. The authors proposed that the incorporation of a discriminator^92^ may promote population diversity and alleviate this shortcoming.

The authors concluded their work by discussing the low synthetic accessibility of most of the generated compounds in all investigated benchmarks and proposed ways of directly accounting for synthesizability in the molecular design process, either during structure generation or fitness evaluation, and using multi-objective GAs^104^ that do not make use of the naive weighted sum approach. In this regard, there are now powerful alternatives like stability filters^34^ and the PL-MOGA,^61^ respectively. The authors furthermore discussed plans for incorporating their previously developed discriminator for population diversity^92^ into the JANUS framework in order to improve the GAs ability to escape local optima.

A combination of the JANUS workflow and discriminator ANNs has been implemented by Nigam and co-workers^34^ for the discovery of organic emitters with inverted singlet-triplet gaps. The discriminator ANNs were trained to identify promising candidates and used as filters to reduce the number of expensive DFT-based fitness evaluations. Furthermore, filters informed by expert opinion were used to remove infeasible structures from the proposed molecules during each generation. With their approach the authors could investigate more than 800 000 molecules and identify at least 10 000 promising emitter candidates.

## Modifying crossover

Constraint handling in GAs is crucial if the design objective entails certain requirements that have to be satisfied and essentially restricts the effective search space by biasing the search towards specific solutions. One common approach for this is to explicitly incorporate appropriate rewards or penalties into the fitness function as a weighted sum. As highlighted in previous works,^92,99^ an important constraint in the evolutionary generation of molecules that is commonly handled this way is the synthetic accessibility accounted for in the penalized log *P* score (eqn (7)). This bears multiple issues however, one of which is the difficulty of choosing appropriate weights to properly balance the optimization goal with rewards and penalties. Furthermore, the weighted sum approach dilutes the fitness value of the actual optimization goal by mixing it with reward or penalty factors. This approach also does not enforce constraints in a strict manner: solutions with both high fitness and low rewards/high penalties can still perform mediocre even though the rewards/penalties are above/below a certain cutoff.

Alternatively, Pareto-based multi-objective optimization techniques can be employed to incorporate the constraints as separate optimization goals. By using appropriate methods to guide the search^61^ effective cutoffs for the constraints can be implemented. However, if there are many constraints to encode or other optimization objectives to consider, optimization efficiency and convergence speed will suffer drastically due to the curse of dimensionality.

An entirely different approach is to modify the genetic operators so that the generated offspring solutions satisfy the desired constraints. In this way, the fitness function remains completely independent from the specific constraints while ensuring that all generated solutions satisfy them intrinsically. Naively, this can be implemented by preselecting proposed offspring solutions based on threshold values for the constraints to be considered.

**Fig. 5:**
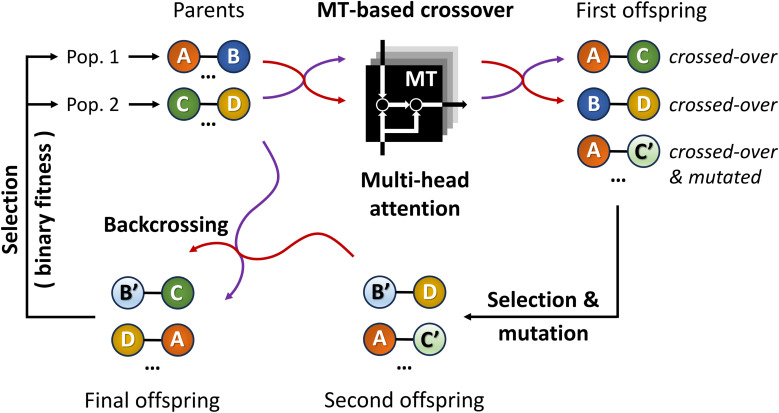
Schematic workflow of the ChemistGA algorithm. Offspring individuals are obtained using the molecular transformer architecture, promoting synthesizability. Backcrossing between the parent and offspring individuals prevents the search from getting stuck in local optima.

By introducing ChemistGA (Fig. 5),^105^ Wang and co-workers demonstrated the use of an ANN-based crossover operation to account for synthetic accessibility during offspring generation in the evolutionary *de novo* design of drug-like compounds. The optimization goals were, in different combinations, protein activities for DRD2, JNK3, and GSK3β, drug-likeness as measured by the QED score,^97^ and synthetic accessibility as measured by the SA score.^93^ All molecules were represented as SMILES strings.

As shown in Fig. 5, the first step in their architecture creates two separate parent populations by randomly drawing a number of solutions from the main population. A crossover operation based on the Molecular Transformer^106^ (MT) ANN is applied to all possible pairs of the two parent populations. The MT is intended to solve the forward problem in synthesis planning by modeling it as a language translation problem: based on two given reactant SMILES, it predicts a product SMILES using a multi-head attention mechanism.^107^ The authors observed that the predicted product inherits structural properties from both reactants and therefore the MT could be implemented as a crossover operation in their GA architecture. Furthermore, because the MT is trained on a dataset of known reactions, it implicitly promotes the synthesizability of the generated molecules. Interesting to note is the fact that the MT is not just a strict crossover between two input individuals since it also introduces entirely new information into the output individuals, by building structures in an autoregressive manner and can therefore be considered a hybrid crossover-mutation operation. The top-50 of all solutions generated in this way are retained and, with a 1% probability, one of these additional mutations is applied: append/insert/delete atom, change atom type/bond order, delete/add ring/bond. These mutations are implemented with SMARTS strings,^108^ which specify molecular substructures using the SMILES line notation. Furthermore, backcrossing between offspring and parents is employed after a certain number of generations by inserting a subset of the initial population into the current population. The purpose of this is to prevent the GA from getting stuck in local optima. Finally, the fitness scores for the generated offspring solutions are calculated and the best performing ones are added to the main population, which is used in the next iteration to again create two separate parent populations. Instead of directly using the continuous fitness values, these are discretized into a binary representation assigning 1 when a prespecified requirement *R* (*e.g.* the SA score^93^ is above a certain threshold) is met or 0 otherwise. The authors claim that this increases the diversity in the selection of individuals.8
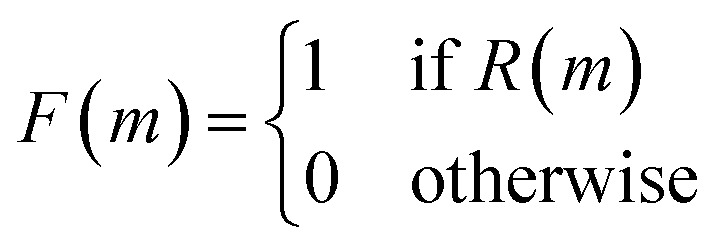


The authors furthermore propose an alternative architecture, R-ChemistGA, that utilizes a random forest surrogate model for molecular property prediction in order to reduce computational cost during fitness evaluation. Every fifth generation the molecular properties are calculated using the true fitness function, and the obtained values are used to retrain the random forest in order to improve the models predictive accuracy. While the resulting model adds noise to the evolutionary process, it is also much more appropriate for a real world application in which property predictions are expensive and may not necessarily be carried out freely.

In a first benchmark experiment, the authors tested their model against GB-GA^67^ on a task aimed at maximizing activity for protein targets JNK3 and GSK3β, as well as the QED and SA scores. Almost 50% of molecules generated by ChemistGA had high activities for both proteins, whereas GB-GA was not able to generate any. Looking at the different optimization goals individually, ChemistGA outperformed GB-GA with respect to all but the SA score, for which the two models performed similarly. Further investigations revealed that ChemistGAs crossover approach facilitated a more reasonable inheritance of molecular patterns due to the MT preserving the parents substructures more accurately.

Next, they investigated performance in terms of synthesizability, novelty, diversity, and the quantity of unique molecular skeleton types based on the Murcko scaffold^109^ for 5000 generated molecules. Two different optimization tasks were assessed: (1) one with only DRD2 and (2) one with JNK3 and GSK3β as protein activity targets in addition to the QED and SA scores. They benchmarked their results against GB-GA and REINVENT.^110^ In both tasks, ChemistGA outperformed the other models in terms of novelty and diversity of generated molecules while achieving similar synthesizability. In terms of the number of generated molecular scaffolds, ChemistGA slightly outperformed REINVENT but was inferior to GB-GA in task (1). In task (2) however, ChemistGA generated more than four times as many distinct scaffolds compared to REINVENT. Overall, the proposed architecture seemed to exhibit higher capabilities of exploring the chemical search space compared to the benchmark models.

Finally, the authors turned their attention to their alternative proposed architecture R-ChemistGA, which uses a random forest surrogate to predict fitness values and ran it on the same two tasks. Compared to their baseline model, ChemistGA, the number of generated molecules with R-ChemistGA exhibiting desired properties was twice as high per evaluation of the true fitness function. This indicates that a GA utilizing a noisy surrogate model is still able to guide the optimization into favorable directions. In terms of synthesizability, diversity, and number of scaffolds, R-ChemistGA performed slightly worse compared to the baseline, though the novelty of generated molecules was the highest of all investigated models. Using a t-SNE^111^ plot of the best synthesizable molecules, the authors furthermore showed that R-ChemistGA explores larger regions of chemical space and asserted that their architecture produced more reasonable molecules with higher synthesizability.

## Evolution in latent space

In applications to chemistry, a key aspect in the design of effective GAs is to find an appropriate chromosomal representation upon which the genetic operators act (crossover and mutation). String representations such as SMILES^51^ and SELFIES^94,95^ are commonly used because they are easy to implement and offer a high degree of flexibility.^38,100,112^ Alternatively, the problem can be discretized by starting from some sort of scaffold that in specific places of the structure allows for the inclusion of molecular fragments chosen from a predefined library.^61,113,114^ A potential issue with this is that the specific representations implicitly define the search space of the EL algorithm and improper choices can limit the search to only certain parts of the chemical space. Therefore, users need domain knowledge in order to make appropriate choices.

**Fig. 6:**
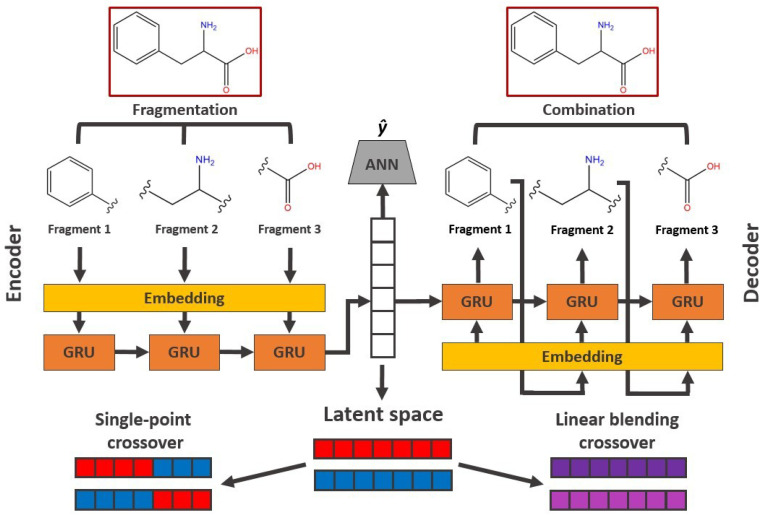
Schematic workflow of the FragVAE architecture that takes molecules in terms of their constituting fragments as inputs and tries to reconstruct them with minimal error. Crossover operations in the EL algorithm operate directly on the latent space representations. The ANN is used to regularize the model with respect to certain properties *ŷ*.

In the Deep Evolutionary Learning (DEL)^115^ framework, Grantham and co-workers made use of an entirely different way of representing molecules in terms of latent space representations learned from autoencoder^116^ type ANN architectures. In particular, they employed a modified variant of a variational autoencoder (VAE)^117,118^ proposed by Podda and co-workers^119^ that operates on molecular fragments in terms of SMILES^51^ strings. Given an input SMILES string, their FragVAE first separates it into fragments and embeds them as tokens using Word2Vec.^120^ In the encoding step of the VAE, the sequence of fragment tokens is passed through gated recurrent units,^121^ encoding them into a latent representation for the full molecule. The decoding step operates in a similar fashion, beginning with a “start of sentence” token and subsequently taking the predicted fragments as inputs to reconstruct molecules similar to the initial input. The VAE is pretrained to minimize the reconstruction loss on an initial training data set, and fine-tuned during evolution on samples from the population. A similar use of a simple VAE in an EL framework had been proposed earlier by Sousa and co-workers.^122^ Grantham and co-workers employed a modification of the original VAE model, first proposed by Gomez and co-workers,^123^ that adds an additional ANN to predict molecular properties from the latent space, which has the effect of regularizing the representations with properties of interest. The EL algorithm was initialized with a random sample from a reference dataset. At the beginning of each generation, the VAE encoder is used to project all individuals in the current population into latent space representations upon which all genetic operators acted directly. Parents are selected based on non-dominated ranking in a multi-objective setting, where individuals are sorted into non-dominated Pareto frontiers. Within each frontier, none of the individuals is better than any other individual with respect to all optimization goals.^124^ Furthermore, the crowding distance, *i.e.* a measures of the density around a particular individual, is employed in order to promote diversity in the population. Two different crossover operations are used: linear blending and single-point^49,50^ crossover. In the former the offspring feature vectors ***z***_1_ and ***z***_2_ are obtained as9
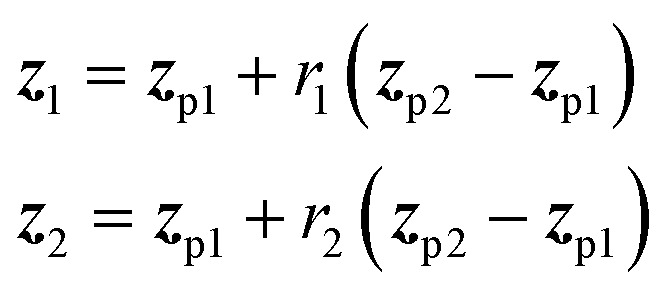
where ***z***_p1_ and ***z***_p2_ denote the latent vector representations of two parents, and *r*_1_ and *r*_2_ are defined as10
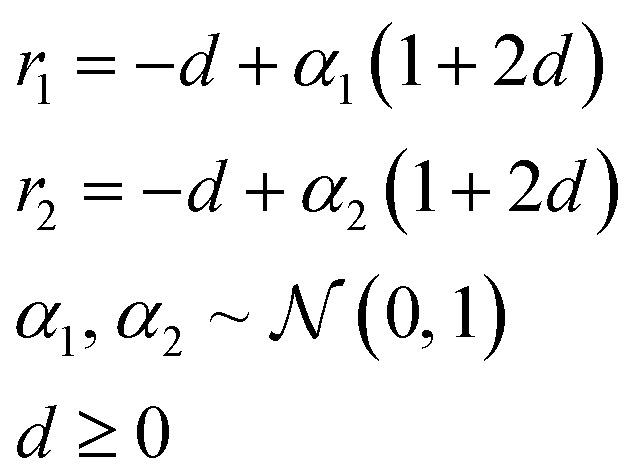
where *d* is a hyperparameter that controls the trade-off between exploration and exploitation, which was set to 0.25 following previous suggestions.^125,126^ The architecture of the FragVAE model including the downstream crossover operations is shown in Fig. 6. After crossover, mutation is randomly applied to all offspring individuals with a probability of 0.01. In the mutations, the representation of offspring individuals is changed in a random single position by adding a normally distributed random variable 
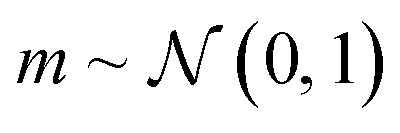
. Next, the decoder part of the VAE is used to generate actual molecular structures from the offspring latent space representations that are then used to determine their fitness. The current population and offspring population are merged together and survivors are subsequently selected in the same way as parents. The new population, including the fitness values, is used to fine-tune the VAE and the next generation is started by projecting the new population into the latent space representations using the VAE encoder. Upon convergence, the algorithm returns the final evolved population.

The authors benchmarked their method on a subset of the ZINC^96^ and PCBA^127^ datasets, simultaneously optimizing the drug-likeness in terms of the QED score,^97^ synthetic accessibility, as measured by the SA score,^93^ and the logarithm of the water–octanol partition coefficient log *P*. Most of the generated molecules were different from the training set and the ratio of unique structures in the final population was high, with values up to 99%. Furthermore, it was reported that the amount of high performing individuals per generation increased along the evolutionary process. By comparing the distributions of the initial data with the distributions over the generated samples, the authors also showed that their approach was able to explore areas of the chemical space beyond the training data. This property of the DEL approach was furthermore highlighted in comparisons with models from the MOSES benchmarking framework,^128^ which in most cases generated distributions very closely aligned with the respective training dataset. In benchmarks against multi-objective Bayesian optimization methods, it was shown that DEL explored a larger hypervolume in the chemical space while also generating molecules of higher performance.

In closing, the authors discussed the scalability and general applicability of their method and proposed the integration of geometric deep learning models to better represent molecules in terms of their 3D structures.

Abouchekeir and co-workers^129^ adapted this approach by replacing the VAE with an adversarial autoencoder (AAE)^130^ that aims at decoding latent space vectors into molecular structures indistinguishable from the training data distribution. Benchmarked on the same datasets and optimization goals, their method produced better candidates in the final population and explored a larger hypervolume in chemical space compared to the original DEL approach. The authors attributed this to the more organized latent space produced by the AAE.

## Summary and outlook

The combination of ML methods and EL strategies for the *de novo* design of molecules and materials is an incipient research topic. However, the here presented studies show that the synergistic interplay of the two paradigms can lead to significant increases in performance. The majority of research in this field seems to focus on surrogate fitness functions that can reduce computational costs by employing ML models to predict fitness values instead of utilizing an expensive reference function. A key technology for the robust exploration of massive chemical spaces are surrogate fitness functions based on Bayesian ML that can acquire new data points on-the-fly. While most efforts so far have focused on Gaussian process regression, future work should explore the applicability of other methods such as Bayesian ANNs.^131–133^ This will allow the research community to more efficiently and accurately explore massive chemical spaces, identifying interesting regions property-wise.

Besides the fitness function, other uses of ML methods in EL seem to be less explored in the literature. However, the works reviewed here for maintaining population diversity^92^ and facilitating constrained crossovers^105^ showed promising results. The proposed models outperformed the GAs that were not augmented with ML, indicating their superior efficiency compared to traditional methods. However, their behavior needs to be further explored on more diverse benchmarks and improved architectures should be derived based on the findings of these investigations. Another interesting perspective can be the use of generative models to produce the molecular fragments used as genes by genetic algorithms.

Most works discussed here only consider single-objective optimization problems or employ some sort of weighted sum approach to condense different optimization goals into one objective. Research on multi-objective GAs that make use of ML surrogate fitness functions seems to be largely unexplored. However, multi-objective evolutionary optimization in chemistry and materials science has many interesting applications with the common goal of efficiently sampling the Pareto front spanned by multiple properties.^61^ Being able to employ surrogate fitness functions in multi-objective settings will also be crucial for enabling the ML accelerated study of these problems. A fundamental question therein is to define the way predictions are facilitated: using a single surrogate model for all objectives or using separate surrogate models, one for each objective. The comprehensive benchmarking of their respective performance in terms of prediction quality and computational cost will contribute to advancing the field, providing researchers with guidelines for choosing the most effective approach to their specific applications.

Another open challenge in this fast evolving field is the comprehensive and unbiased benchmarking of proposed methods across diverse domains. Many of the works reviewed here report promising performances of their proposed ML-augmented GA architectures but only partially tested against classical alternatives. For example, the discriminator ANNs for ensuring population diversity introduced by Nigam and co-workers^92^ was only benchmarked against a standard GA architecture but not one employing classical techniques for maintaining diversity such as niching.^124,134^ Similarly, ChemistGA^105^ was only benchmarked against a very narrow set of classical GAs not taking into account a wider range of heuristic crossover operations and using suboptimal parameters. Future work should put emphasis on fair and informative benchmarking comparisons against relevant baselines. Especially when comparing the computational efficiencies of classical and ML-augmented GAs, care should be exercised to also take potential training and evaluation costs of ANNs into account. Furthermore problematic is the fact that some of the commonly employed benchmarks such as GuacaMol^98^ and MOSES^128^ have become outdated since modern EL methods are able to consistently obtain near-perfect scores on them^101,112,135^ making it difficult to make substantiated statements about their respective performances. In this regard, Nigam and co-workers developed Tartarus,^32^ a suite of realistic benchmarking sets entailing molecular design tasks from chemistry and materials science. In total, the study introduces four novel benchmarks across diverse domains that each include curated reference datasets. The authors encourage employing a resource-constrained training approach limiting the overall runtime and the number of proposed candidates in order to ensure the meaningful comparison of different methods.

An interesting ML-based modification of GAs that has not been addressed in the literature, are the so-called Δ-ML approaches for fitness surrogate functions. In Δ-ML, instead of directly trying to predict the ground truth, a correction term to a cheap approximation to the reference method is learned. The final prediction can then be obtained with

11 *y*^ref^ = *y*^approx^ + *Δ*

where *y*^ref^ denotes the ground truth as defined by the reference method, *y*^approx^ denotes an approximation to the ground truth, and *Δ* denotes the learned correction between both. Because this approach requires the additional evaluation of an approximation method, it is has a higher computational cost than standard ML. However, Δ-ML approaches bear the advantage that the features used to predict the correction term can come from the approximation method. These features might contain more relevant information that can be leveraged in the ML model to reduce errors. Overall, research suggests a significant increase in predictive performance.^136–138^

In chemistry and materials science, an interesting application for Δ-ML is the prediction of corrections for energies and properties from semiempirical approximations^139–141^ such as GFN2-xTB^142^ to *ab initio* methods such as DFT. In evolutionary molecule design, fitness functions based on *ab initio* calculations are often times associated with prohibitively high computational costs, whereas semiempirical approximations are usually feasible. The use of Δ-ML in EL applications as a cheap yet accurate fitness function can potentially lead to better convergence properties and increase the quality of the solutions evolved.

Finally, the complex nature of such synergistic architectures requires users to have extensive knowledge of evolutionary and machine learning, rendering them difficult to use by non-experts. Efforts should go into the development of general frameworks that make these methods more easily accessible by a larger community, in order to enable their application to interesting problems within the fields of chemistry and materials science. For this, a culture of open code and data is crucial in which support for command line usage and comprehensive documentation facilitates the use and adaptation of existing methods. Furthermore, promoting avid exchange between method developers and users, as well as between the theoretical and experimental communities, will help to increase the scientific impact of these methods.

## Author contributions

HK was the main contributor to the writing of the manuscript, whereas DB was the main contributor to its revision. Both authors contributed to the scientific discussions defining the contents of this perspective.

## Conflicts of interest

There are no conflicts to declare.

## Data Availability

The data availability statement is not relevant to this manuscript since it is a perspective article which does not report any new data.
